# Eagle’s Syndrome: A Fortunate Discovery in a Symptomatic Patient

**DOI:** 10.7759/cureus.61181

**Published:** 2024-05-27

**Authors:** Vaya R Karapepera, Anna Aourelia Maria Skarmoutsou, Dimitrios Tziouris

**Affiliations:** 1 Otolaryngology - Head and Neck, Chatzikosta Hospital Ioannina, Ioannina, GRC; 2 1st General Surgery Department, University Hospital of Alexandroupolis, Alexandroupolis, GRC; 3 Otolaryngology - Head and Neck Surgery, Chatzikosta Hospital Ioannina, Ioannina, GRC

**Keywords:** throat pain, neck pain, styloid process, otorhinolaryngology, eagle's syndrome

## Abstract

Eagle’s syndrome is a condition characterized by an elongated styloid process or a calcified stylohyoid ligament, which can lead to a plethora of symptoms, such as neck and facial pain upon movement, dysphagia, pharyngeal foreign body sensation, headache, and vertigo-like sensations. This pathology may affect one or both of a patient's styloid processes (unilateral or bilateral), with most of these cases going undiagnosed due to the vague nature of their symptoms. Nonetheless, the diagnosis of Eagle’s syndrome must derive from the combined findings of both clinical examination and radiographic imaging. Symptomatic patients may require conservative or surgical treatment.

## Introduction

On average, in adults, the styloid process’ length is approximately 2.5 cm with its tip being located between the external and internal carotid arteries. Thus, any process longer than 2.5 cm is considered abnormally elongated [1,2,3]. Such styloid processes, whether bilateral or unilateral, may interfere with the functioning of neighboring anatomical structures, causing a wide range of symptoms that constitute Eagle’s syndrome, first described by Watt W. Eagle in 1937 [4,5]. The incidence of an elongated styloid process is reported as 4% of the general population. However, only 4% of these cases are symptomatic, and therefore, the true incidence of Eagle’s syndrome is about 0.16%, showing a three times higher prevalence in females compared to males. Patients are usually older than 30 years of age, and they usually have bilaterally elongated processes (although unilateral cases have also been described) [6,7].

Because of its rarity and the vague or atypical symptoms, only one in 10,000 patients with Eagle’s syndrome are clinically recognized. The majority of the symptoms related to Eagle’s syndrome are nonspecific and include orofacial and cervical pain, dysphagia, foreign body sensations, and even dizziness upon neck movement. This is why they are often falsely attributed to a wide variety of other pathologies. Differential diagnoses include migraine-type headaches, facial neuralgias or oral, dental, and hyoid bursitis, otitis, temporomandibular diseases, temporal arteritis, cervical arthritis, carotid artery dissection, glossopharyngeal neuralgia, head and neck tumors, cervical mass, faulty dental prostheses, esophageal diverticula, salivary gland disease, and trigeminal neuralgia [5,7,8,9]. Therefore, due to the commonality of these symptoms and the rarity of Eagle syndrome, our main insight from this case is the importance of physician awareness of the syndrome to ensure accurate diagnosis.

We report a case of bilaterally elongated styloid processes to more than 60 mm in a 56-year-old female patient, which was an incidental finding.

## Case presentation

**Figure 1:**
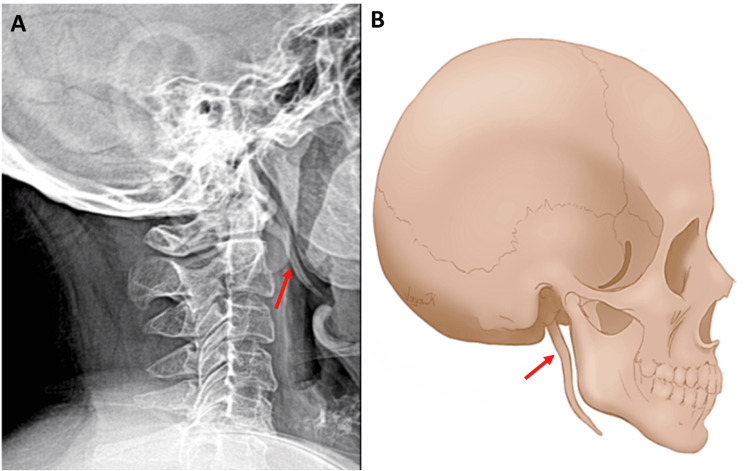
A) X-ray of the patient showing elongated styloid processes and B) explanation drawing. In both 1A and 1B, the arrows point to elongated styloid processes.

A previously healthy 56-year-old woman visited the emergency department of our hospital due to shoulder pain after stumbling and falling on the right side of her body. Her initial complaints included mild right shoulder and right abdominal pain, without mentioning any head injury. The patient’s past medical and medication history was unremarkable. However, the patient described a decade-old chronic facial and neck pain triggered by head movements. Initial vital signs included a blood pressure of 123/85 mmHg, heart rate of 85 beats per minute, and oxygen saturation of 98%. The first assessment revealed slight sensitivity of the patient’s right shoulder and the incidental finding of head and neck sensitivity upon palpation of the neck area on the upper border of the sternocleidomastoid muscle and behind the mandibular angle bilaterally. Radiographic imaging showed no fractures or dislocation of the shoulder, no signs of thoracic or abdominal injury, and no signs of vertebral or head injury, with blood tests within normal ranges. However, X-rays of the head and cervical vertebrae revealed the incidental finding of bilaterally elongated styloid processes, extending downward below the angle of the mandible in the lateral view (Figure 1).

The patient’s chronic pain symptoms had never been attributed to this finding before. During clinical examination and palpation, the stylohyoid ligament could not be felt either posteriorly to the mandible or inside the oral cavity. Nonetheless, radiographic measurements determined their length to be 60 mm. Following these findings, a thorough otorhinolaryngological and neurological examination was performed, ruling out other possible pathologies including otitis, vertigo, trigeminal neuralgia, and migraines. No dental problems were found, and a brain CT scan excluded any possible brain pathology or neoplastic etiology. Considering the clinical and radiographic findings, we set the diagnose of Eagle’s syndrome. The patient received conservative therapy for symptom alleviation and conservative orthopedic care for her shoulder pain. Specifically, the treatment plan included gradually reducing dosages of paracetamol, followed by a week of similarly reduced dosages of ibuprofen alternating with intervals of no medicine intake. At the one-year follow-up examination, it was determined that the patient’s symptoms were sufficiently managed with conservative treatment. The patient was fully satisfied with the results and reported that her symptoms were now mostly nonexistent or extremely mild during different time periods.

## Discussion

Many hypotheses have been made regarding the pathophysiology of Eagle‘s syndrome. Dr. Watt Eagle first examined the possible causes of this interesting pathology and suggested that the elongated styloid process is the result of reactive ossification of the stylohyoid ligament, due to inflammation. Such inflammatory conditions are thought to be osteitis, local chronic irritation, periostitis or tendonitis of the styloid process and the stylohyoid ligaments, and surgical trauma. Specifically, it has been described that after tonsillectomy, scar tissue develops under the tonsillar fossa, compressing and stretching cranial nerves V, VII, IX, and X. This reaction results in the development of Eagle’s syndrome [4,10,11].

Moreover, Lentini (1975) proposed that, after a stressful inflammatory or traumatic event, persistent mesenchymal elements (Reichert cartilage residues) often undergo osseous metaplasia [4]. Later, Epifanio (1962) speculated that another factor causing ossification of the stylohyoid ligament may be hormonal imbalances in menopausal women since they also had multiple ossified ligaments in various parts of their bodies [4]. We determined that this hypothesis could correlate to this case since our patient is a 56-year-old female who had experienced menopause since the age of 46. However, her medical history revealed no endocrine disorders. Gokce et al. (2008) proposed that conditions, such as end-stage renal disease, hypercalcemia, and vitamin D metabolism disorders, lead to calcification of the styloid ligament and, thus, elongated styloid processes [4]. Lastly, Sekerci (2015) explored the relationship between the presence of an arcuate foramen and an elongated styloid process and found a correlation between the two [10,11]. The majority of patients with Eagle syndrome present with a constant dull pharyngeal pain focused in the ipsilateral tonsillar fossa. This discomfort may radiate to the ear and worsen with head rotation, mirroring the symptoms reported by our patient. However, in some rarer cases, the elongated styloid process can cause episodic tic-like pain attacks, typical of glossopharyngeal neuralgia. In addition, as mentioned previously, other symptoms include otalgia, headache, painful swallowing, pain along with the distribution of the external and internal carotid arteries, the sensation of a foreign body in the pharynx, and facial pain. Finally, a hard mass may be palpated below the angle of the mandible, reproducing the patient’s symptoms [4,12,13]. In our case, no mass was palpable intraorally or externally.

Various potential mechanisms have been suggested for the pathogenesis of pain as a symptom of Eagle‘s syndrome. First, it is speculated that the enlarged styloid process may cause pressure to several nerves in the neck area, most commonly the glossopharyngeal nerve, which contributes to painful throat sensation. Moreover, it has been hypothesized that the styloid process may cause compression to the internal carotid artery, which leads to momentary ischemic episodes upon movement or excess pressure to the sympathetic plexus, subsequently causing pain to these specific areas of the neck [12,13]. Cases of Eagle’s syndrome that include these symptoms fall into the category of "carotid artery type." These may also manifest with additional symptoms, such as migraine-type headaches, temporal headaches, ipsilateral headaches, vertigo, and neurological manifestations caused by irritation of the sympathetic plexus, and even syncope, triggered particularly during head movements. [9,14]. In addition, pain may not always be the result of direct pressure from the elongated styloid process, but it may also derive from compression of nerve endings inside the tonsillar tissue due to scarring [6].

The identification of Eagle’s syndrome typically relies on a thorough medical history and physical examination. The nonspecific symptoms that characterize this condition (such as orofacial pain, dysphagia, and dizziness upon neck movement) could stem from a variety of other pathologies, as mentioned before. Therefore, before arriving at a diagnosis, other possible conditions should be ruled out, as we did in our case. During clinical examination, intraoral palpation in the tonsillar fossa can detect an elongated styloid process. If pain triggered by palpation spreads to the ear, face, or head, it suggests that an elongated styloid process is probable as the underlying cause. A normal-length styloid process typically remains undetectable or imperceptible through palpation. The diagnosis of the syndrome should then be confirmed by imaging [15].

There are two main treatment approaches for managing elongated styloid process syndrome: conservative or surgical. Conservative methods may include analgesics, antidepressants, lidocaine, anticonvulsants, nonsteroidal anti-inflammatory drugs, or the application of topical heat. Furthermore, transpharyngeal injection of steroids and lidocaine and the use of diazepam are also considered conservative options [14,15,16]. Clinicians should prioritize exploring conservative treatments before considering surgical options. However, if a patient does not respond positively to multiple medications, they may require surgical intervention. The preferred surgical method is shortening the styloid process through an intraoral or extraoral approach, as it typically yields improved long-term outcomes [14,15,16]. In the present case, the patient exhibited responsiveness to oral analgesic medications, obviating the necessity for surgical intervention.

## Conclusions

Eagle’s syndrome is a rare but often misdiagnosed condition due to the vague and nonspecific nature of its symptoms, which can easily be attributed to other conditions, such as migraine-type headaches, facial or dental neuralgias, otitis, temporomandibular diseases, vertigo, and even tumors. A comprehensive physical examination is essential to exclude these alternative pathologies. The diagnosis should be based on both a detailed patient history, thorough clinical assessment, and radiological imaging. In this case report, we describe a patient with chronic facial and neck pain triggered by head movements who underwent a thorough physical examination, clinical assessment, and radiological imaging, ultimately leading to a diagnosis of Eagle’s syndrome. Our patient responded well to treatment with oral analgesics, and surgical intervention was unnecessary. We believe that although this condition is rare, it is crucial for clinicians to be aware of such abnormalities to ensure successful recognition and treatment.
